# The ameliorative effects of sesamol against seizures, cognitive impairment and oxidative stress in the experimental model of epilepsy

**Published:** 2014-02

**Authors:** Parichehr Hassanzadeh, Elham Arbabi, Fatemeh Rostami

**Affiliations:** 1 Iranian Center of Neurological Research, Tehran University of Medical Sciences, Tehran, Iran; 2 Research Center for Gastroenterology and Liver Diseases, Shahid Beheshti University of Medical Sciences, Tehran, Iran

**Keywords:** Cognitive impairment, Epilepsy, Kindling, Oxidative stress, Rat, Sesamol

## Abstract

***Objective(s):*** A growing interest has recently been attracted towards the identification of plant-based medications including those with protective effects against cognitive impairment. Sesamol has shown promising antioxidant and neuroprotective effects, therefore, we aimed to evaluate its therapeutic potential in epilepsy which is commonly associated with oxidative stress and cognitive impairment.

***Materials and Methods:*** Male Wistar rats received pentylenetetrazole (PTZ) (30 mg/kg, IP) once every other day until the development of kindling, i.e., the occurrence of stage 5 of seizures for three consecutive trials. After the completion of kindling procedure, behavioural tests including elevated plus maze and passive avoidance were performed in order to assess *learning and memory*. Oxidative stress was assessed by estimation of lipid peroxidation and reduced glutathione. The effects of pretreatment with sesamol (10, 20, and 30 mg/kg, IP) against PTZ-induced seizures, *cognitive* *impairment* and *oxidative* *stress* were investigated.

***Results:*** 32.45 ± 1.86 days after treatment with PTZ, kindling was developed that was associated with myoclonic jerks and generalized tonic-clonic seizures. Moreover, PTZ kindling induced a remarkable cognitive impairment and oxidative stress. Sesamol (30 mg/kg) significantly delayed the development of kindling and prevented seizure-induced cognitive impairment and oxidative stress.

*Conclusion:* Sesamol exerts ameliorative effects in the experimental model of epilepsy. This phytochemical may be considered as a beneficial adjuvant for antiepileptic drugs.

## Introduction

In recent years, a growing interest has been attracted towards the complementary and altern-ative medicines including the plant-based medica-tions. Plants, similar to conventional medicines, are able to synthesize a wide variety of biologically active compounds which exert their effects through the interaction with biological pathways (1). Sesamol, the major constituent of sesame seed oil (obtained from *Sesamum indicum*, Linn, Pedaliaceae), is a traditionally used health supplement which exerts antioxidant, anti-inflammatory, immunomod-ulatory, neuroptotective, anti-aging, chemopreve-ntive, and antidepressant effects (2-9). In the experimental model of diabetes, sesamol suppressed neuro-inflammatory cascade, improved the function of blood-brain barrier, and prevented cognitive deficits (10-12). Moreover, sesamol has shown a protective effect against cognitive dysfunction induced by 3-nitropropionic acid as well as free radical scavenging activity (13, 14). We therefore hypoth-esized that sesamol might exert therapeutic potential in epilepsy, a syndrome with divergent symptoms involving excessive neuronal activity in brain and numerous seizures. This chronic neurological dis-order is associated with the generation of reactive oxygen species and cognitive impairment, as a large number of patients with epilepsy suffer from learn-ing and memory deficits (15). Hypoxic ischemic encephalopathy, CNS infections or trauma, strokes, brain cancer, cerebrovascular diseases, and drug or alcohol abuse may contribute to the ethiopatho-genesis of epilepsy. Epileptic seizures may also occur in recovering patients as a consequence of brain surgery (16). Epilepsy is usually controlled, but not cured, by anticonvulsant medications. However, seizure are not controlled by medications in more than 30% of patients, hence, the neurosurgical operations may be considered as an alternative therapy in order to reduce the frequency or severity of seizures (17). Moreover, antiepileptic drugs curr-ently in use are associated with limited efficiency and a significant number of side effects (18, 19).

In the present study, we evaluated the effect of sesamol on seizures following pentylenetetrazole (PTZ)-induced kindling, a widely used experimental model for the development of seizure and estim-ation of the effectiveness of antiepileptic drugs. During kindling epileptogenesis, the brain is repeatedly stimulated that initially lowers the seizure threshold leading to the occurrence of seizures (20). The gradually developed seizures result in the generalized tonic-clonic seizures, neur-onal loss in brain regions including the hippocampus and amygdala, and cognitive impair-ment (21). In other parts of the study, the effects of sesamol on the probable cognitive impairment and oxidative stress induced by PTZ kindling were investigated.

## Materials and Methods

***Animals***

Male Wistar rats weighing 250-280 g from our institution’s laboratory animal centre were randomly assigned and housed three per cage under standard laboratory conditions with a 12-hr light/dark cycle. Food pellets and water were available *ad libitum*, however, animals were deprived of food 12 hr before the behavioural assessment. Experimental proced-ures were approved by the local Ethics Committee. 

***Treatments***

PTZ (Sigma Aldrich, Germany) was dissolved in 0.9% saline and injected intraperitoneally (IP) at dose of 30 mg/kg once every alternate day (48±2 hr) until the development of stage 5 of seizures for three consecutive trials (22) (n=6/group). In other cohort groups of animals, sesamol (3,4-methylenedioxy-phenol), (Sigma Aldrich, Germany) was dissolved in 0.5% dimethyl sulfoxide (DMSO, Sigma Aldrich, Germany) and administered IP at doses of 10, 20, and 30 mg/kg (13, 23) 30 min prior to the injection of PTZ (n=6/group). PTZ and sesamol were freshly prepared throughout the study. C*ontrol groups* *received the corresponding vehicle* solutions (n=6/group). Injections were performed between 9:00 a.m. and 10:00 a.m. and the injection volume was 1 ml/kg. 

***PTZ kindling***

After each injection of PTZ, animals were observed for 30 min in order to assess the seizure activity using the following scale; stage 0: no response, stage 1: hyperactivity, vibrissae twitching, stage 2: head nodding, head clonus and myoclonic jerks, stage 3: unilateral forelimb clonus, stage 4: rearing and bilateral forelimb clonus, stage 5: generalized tonic-clonic seizure (GTCS) and loss of writing reflex (24). Animals exhibiting stage 5 of seizures for three consecutive trials were considered kindled (22). Time required for the development of kindling, numbers of myoclonic jerks and duration of GTCS were recorded. 

***Behavioural tests***

After the completion of kindling procedure, behavioural tests were performed (n=6/group). Only one rat was tested at a time.

***Elevated plus-maze test***

Spatial learning was evaluated using the elevated plus-maze test as previously described (25, 26). The apparatus consisted of two open arms (50 × 10 cm) surrounded by a short (1 cm) plexiglass edge to prevent falls and two enclosed arms (50 × 10 × 40 cm) extended from a central platform (10 × 10 cm). The height of the maze was 40 cm above the floor. The maze was cleaned thoroughly with alcohol-water solution after the removal of each rat in order to remove any confounding olfactory cues. The acquisition session was performed on day 1 during which each rat was gently placed at the distal end of an open arm of the apparatus and the time taken for animal entrance (with all four limbs) to any one of the closed arms was recorded as the initial transfer latency. Each rat was allowed to move freely for 10 sec when it entered the closed arm. Those animals that did not enter the closed arms within the cut off time (90 sec), were excluded from the study. Following the acquisition session, the animal was returned to its home cage. 24 hr after the acquisition session (day 2), the test of retention transfer latency was performed in a similar manner as the acquisition trial. 

***Passive avoidance test***

A one-trial, step-through, light-dark passive avoidance apparatus (Ugo Basile model 7551, Comerio, Italy) was used for the evaluation of emotional memory based on the contextual fear conditioning learning (27, 28). The reduction of step-through latency was used as an indicator of impaired memory. The apparatus consisted of two illuminated and dark chambers which were separated by a flat-box partition including an automatically operated sliding door. The dark chamber was equipped with an electrifiable grid floor. A pre-acquisition trial was performed on the first day of training in which rats were placed individually in the illuminated chamber and allowed to explore the apparatus for 3 min. The door between the two chambers was opened after 30 sec and the animal was able to move freely into the dark chamber. Fifteen min after the pre-acquisition trial, an acquisition trial was performed and each animal was placed in the illuminated chamber. Following 60 sec of habituation, the door between the chambers was opened and the initial latency for animal entrance (with all four limbs) into the dark chamber was recorded. Animals showing the initial latency time more than 60 sec were excluded from further experiments. As soon as the animal entered the dark chamber, the sliding door was closed automatically and an electrical shock through the grid floor was delivered to the animal’s feet via a shock generator (0.5 mA for 3 sec). The animal was then removed from the dark chamber and returned into its home cage. Between each training session, both of the chambers were cleaned thoroughly in order to remove any confounding olfactory cues. 24 hr after the acquisition trial, a retention trial was performed in the same way as aforementioned, however, the animals did not receive foot-shock. The latency time was recorded up to maximum 600 sec. 

**Figure 1:**
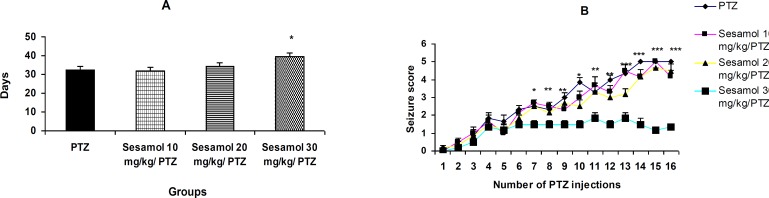
The effect of sesamol on the development of kindling. A: time required for the development of kindling; sesamol delayed the development of kindling in a dose-dependent fashion. B: seizure score; pretreatment with 30 mg/kg sesamol significantly reduced the seizure scores. Data are represented as mean ± SEM (n=6/group).

(PTZ: pentylenetetrazole, Sesamol .../ PTZ: administration of sesamol before the injection of PTZ)

A:^ *^ *P*<0.05 *vs* PTZ group, B: ^*^ *P*<0.05, ^**^ *P*<0.01, ^***^ *P*<0.001 *vs* sesamol 30 mg/kg/PTZ

***Biochemical tests***

Following the behavioural tests, animals were killed by decapitation and their brains were quickly removed, rinsed with ice-cold saline and stored at -70 ° C for further analysis. Just before the evaluation of oxidative stress, brain tissue samples were thawed and 10% (w/v) homogenates were prepared by ice-cold 0.1 M phosphate buffer (pH 7.4). Aliquots of homogenates were used to assess lipid peroxidation and reduced glutathione. 

***Evaluation of lipid peroxidation***

Malondialdehyde (MDA) content was determined as previously described (29). MDA, an end product of lipid peroxidation, reacts with thiobarbituric acid (TBA) to form a pink-coloured chromophore. Measurement of MDA content by TBA reactivity is the most widely used method to assess lipid peroxidation. In brief, 1.5 ml of 20% (v/v) acetic acid (pH 3.5), 1.5 ml of 0.8% (w/v) TBA (Sigma Aldrich, Germany) and 0.2 ml of 8.1% (w/v) sodium dodecyl sulphate (Sigma Aldrich, Germany) were added to 0.1 ml of processed brain tissue sample and heated at 95°C for 60 min. The mixture was cooled, vortexed, and centrifuged at 4000 rpm for 10 min. Absorbance of the resultant supernatant was measu-red at 530 nm using a spectrophotometer (UV-1601, Shimadzu, Japan). The measurements were perform-ed in duplicate and analyzed by an investigator blind to the experimental set-up. The concentration of MDA is expressed as nM/g wet tissue weight.

***Measurement of reduced glutathione***

Reduced glutathione (GSH) was measured as previously described (30). Briefly, 1 ml of homog-enate was mixed with 10% trichloroacetic acid (Sigma Aldrich, Germany) and centrifuged at 5000 rpm for 10 min. Then, 2 ml of 0.3 M phosphate buffer (pH 8.4), 0.5 ml of 5’5-Dithiobis (2-nitrobenzoic acid) (Sigma Aldrich, Germany) and 0.4 ml of double distilled water were added to 0.1 ml of supernatant. The mixture was vortexed and absorbance was measured at 412 nm. The measurements were perfo-rmed in duplicate and analyzed by an investigator blind to the experimental set-up. The concentration of GSH is expressed as µg/g wet tissue weight.

***Statistical analysis***

The Kolmogorov-Smirnov test was used to verify normal distribution of the experimental data. Repeated measures ANOVA was applied to evaluate the development of seizures in the course of kindl-ing. One-way ANOVA followed by Tukey’s *post hoc *test was used for the analysis of behavioural and biochemical data. Results are expressed as mean ± SEM (six animals per group). The level of signify-cance was set at *P*<0.05.

## Results

***The effect of sesamol on the development of kindling***

PTZ induced kindling after 32.45 ± 1.86 days (Fiugure 1A). Sesamol at dose of 30 mg/kg significantly delayed the development of kindling as compared to PTZ group (Figure 1A, *P*<0.05), while, the lower dos-es were ineffective (Figure 1A, *P*>0.05). Pretreatment with 30 mg/kg sesamol significantly reduced the seizure scores (Figure 1B, *P*<0.05, *P*<0.01, and *P*<0.001).

**Figure 2:**
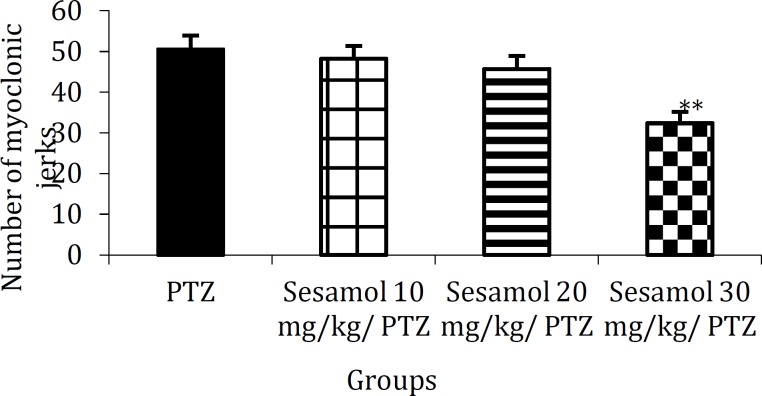
The effect of sesamol on the number of myoclonic jerks in kindled rats. Sesamol dose-dependently reduced the number of PTZ-induced myoclonic jerks. Each column represents mean ± SEM (n=6/group).

^**^ *P*<0.01 *vs* PTZ group

***The effect of sesamol on the number of myoclonic jerks in kindled rats***

The number of myoclonic jerks in PTZ group (Figure 2, 50.5 ± 3.35) was remarkably decreased due to the pretreatment with 30 mg/kg sesamol (Figure 2, 32.13 ± 2.79, *P*<0.01). 

***The effect of sesamol on the duration of GTCS in PTZ group***

**Figure 3:**
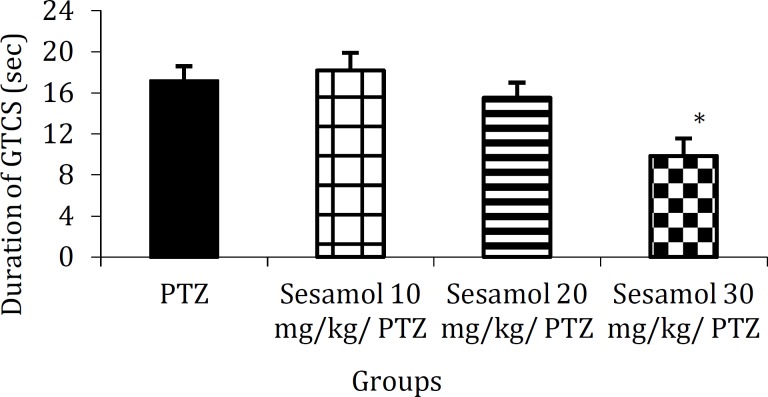
The effect of sesamol on the duration of generalized tonic-clonic seizure in pentylenetetrazole group. Pre-application of sesamol at the highest dose tested reduced the duration of GTCS in kindled rats. Each column represents mean ± SEM (n=6/group).

^*^ *P*<0.05 *vs* PTZ group (sec: second, GTCS: generalized tonic-clonic seizure)

Pretreatment with 30 mg/kg sesamol signify-cantly reduced the duration of GTCS as compared to PTZ group (Figure 3, *P*<0.05).

***The effect of sesamol on PTZ-induced cognitive impairment using elevated plus-maze***

**Figure 4:**
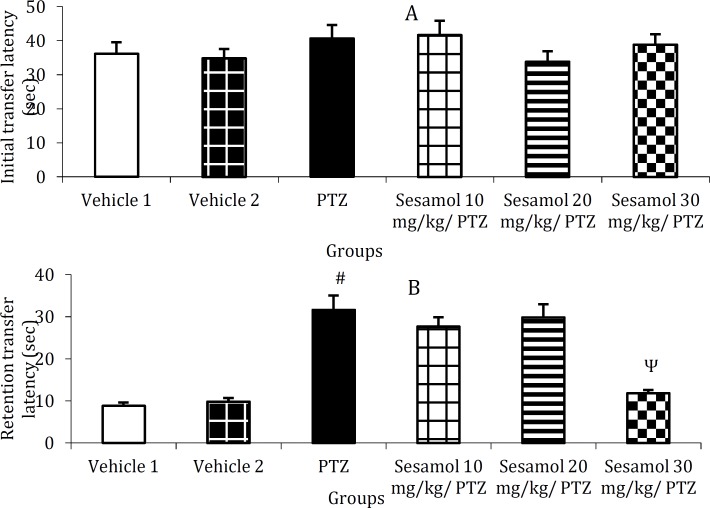
The effect of sesamol on pentylenetetrazole-induced cognitive impairment using elevated plus-maze. A: initial transfer latency, B: retention transfer latency. Each column represents mean ± SEM (n=6/group). Vehicles 1 and 2 are related to PTZ and sesamol, respectively.

^#^ *P*<0.001 *vs* vehicle 1, ^Ψ^ *P*<0.001 *vs* PTZ group and the groups pretreated with 10 and 20 mg/kg sesamol

In the initial transfer latency, there was no significant difference between the groups (Figure 4A, *P*>0.05), while, retention transfer latency was remarkably elevated in PTZ group as compared to the control group (Figure 4B, *P*<0.001). Following the pretreatment with sesamol (30 mg/kg), the retention transfer latency was significantly reduced as compared to PTZ group and the groups pretreated with 10 and 20 mg/kg sesamol (Figure 4B, *P*<0.001) and remained at control level (Figure 4B, *P*>0.05). 

***The effect of sesamol on PTZ-induced cognitive impairment using passive avoidance test***

**Figure 5:**
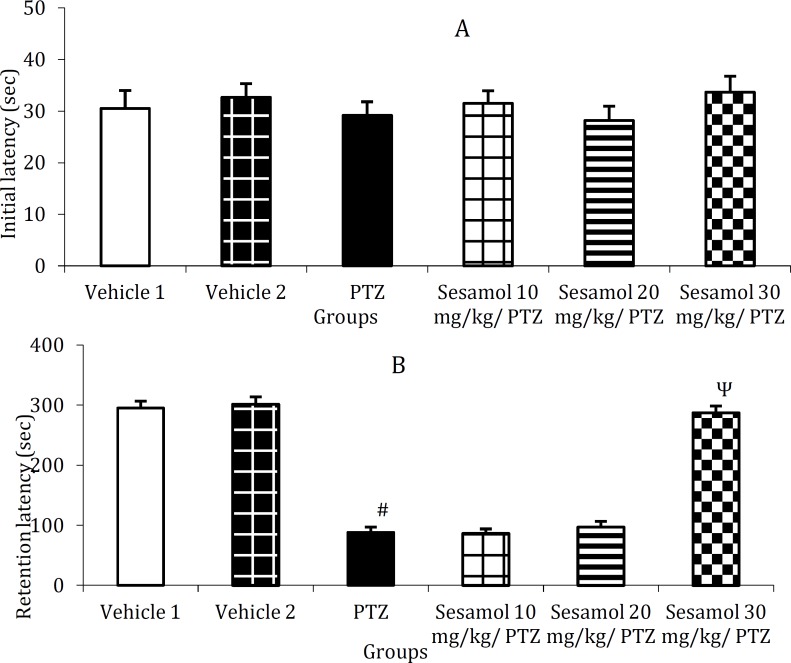
**The **effect of sesamol on pentylenetetrazole-induced cognitive impairment using passive avoidance test. A: initial latency, B: retention latency. Each column represents mean ± SEM (n=6/group).

^#^ *P*<0.001 *vs* vehicle 1,^ Ψ^ *P*<0.001 *vs* PTZ group and the groups pretreated with 10 and 20 mg/kg sesamol

The mean initial latency did not significantly differ between the groups (Figure 5A, *P*>0.05), 

however, the retention latency was remarkably reduced in PTZ group as compared to the control group (Figure 5B, *P*<0.001). Following the pretreatment with 30 mg/kg sesamol, the retention latency was significantly increased as compared with PTZ group and the groups pretreated with 10 and 20 mg/kg sesamol (Figure 5B, *P*<0.001) and remained at control level (Figure 5B, *P*>0.05).

***The effect of sesamol on brain MDA level in kindled rats***

**Figure 6:**
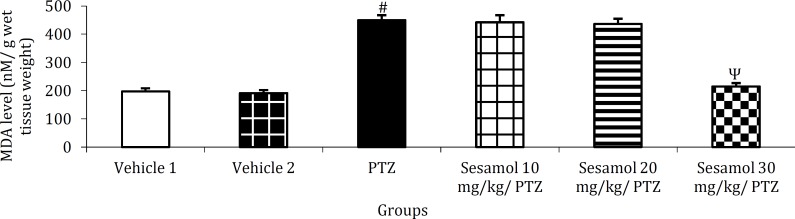
The effect of sesamol on brain malondialdehyde level in kindled rats. Pre-application of sesamol at the highest dose tested prevented PTZ-induced enhancement of brain MDA content. Each column represents mean ± SEM (n=6/group).

^#^ *P*<0.001 *vs* vehicle 1, ^Ψ^ *P*<0.001 *vs* PTZ group and the groups pretreated with 10 and 20 mg/kg sesamol

PTZ kindling resulted in a significant enhance-ment of brain MDA level as compared to the vehicle group (Figure 6, *P*<0.001). This, was prevented by the pretreatment with 30 mg/kg sesamol (Figure 6, *P*<0.001 *vs* PTZ group and the groups pretreated with 10 and 20 mg/kg sesamol). 

***The effect of sesamol on brain GSH content in kindled rats***

**Figure 7:**
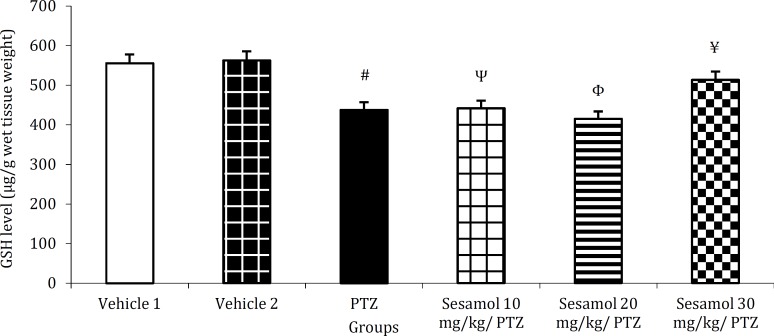
The effect of sesamol on brain glutathione content in kindled rats. Sesamol dose-dependently preserved brain GSH at control level. Each column represents mean ± SEM (n=6/group).

^#^ *P*<0.01 *vs* vehicle 1, ^Ψ^ *P*<0.01 *vs* vehicles 1 and 2, ^Φ^ *P*<0.001 *vs* vehicles 1 and 2, ^¥^ *P*<0.05 *vs* PTZ group and the group pretreated with 10 mg/kg sesamol, and *P*<0.01 *vs* the group pretreated with 20 mg/kg sesamol

In PTZ-treated rats, brain GSH content was significantly lower as compared to the vehicle group (Figure 7, *P*<0.01). GSH level remained significantly lower than vehicle groups following the pretreat-ment with 10 or 20 mg/kg sesamol (Figure 7, *P*<0.01 and *P*<0.001, respectively). GSH content remained at control level following the pretreatment with 30 mg/kg sesamol (Figure 7, *P*>0.05) and was significa-ntly higher than PTZ group (Figure 7, *P*<0.05) and the groups pretreated with 10 and 20 mg/kg sesamol (Figure 7, *P*<0.05 and *P*<0.01, respectively).

## Discussion

In recent years, herbal medicines including those exerting antioxidant and neuroptotective effects have become the focus of intense research. Sesamol is a traditionally used health supplement which ex-erts various cellular effects including antioxidant and neuroprotective effects and has multiple biological targets (2-14). In the present study, we have evalu-ated the therapeutic potential of sesamol against one of the major neurological disorders; epilepsy. Using an experimental model of epilepsy, we found that repeated administration of PTZ (30 mg/kg) on alternate days resulted in the enhancement of convu-lsive activity and stage 5 of seizures for three consecutive trials (Figure 1). Sesamol dose-dependently delayed the development of PTZ-indu-ced kindling (Figure 1) and reduced the number of myoclonic jerks (Figure 2) and duration of GTCS in kindled animals (Figure 3). These findings suggest the preventive and ameliorative effects of sesamol against PTZ-induced seizures. 

(nM: nanomolar, MDA: malondialdehyde)

(µg: mcg, GSH: reduced glutathione)

As shown in Figure 4B, PTZ treatment resulted in a significant increase of retention transfer latency in the elevated plus maze test. In addition, retention latency was remarkably reduced in PTZ-treated animals in passive avoidance paradigm (Figure 5B). These data indicate the occurrence of cognitive impairment in kindled rats. Our findings are in agreement with previous reports indicating the deteriorative effect of kindling on learning and memory (21, 31). Several factors including persisting seizures and degenerative processes in brain regions which may be secondary to seizure-related hypoxia and ischemia may contribute to cognitive impair-ment (19, 31). Following the pretreatment with sesamol (30 mg/kg), the retention transfer latency was significantly reduced in the elevated plus maze test (Figure 4B). Moreover, sesamol dose-depend-ently reversed the reduction of retention latency in PTZ-treated group during passive avoidance test (Figure 5B). These data show the protective effects of sesamol against the cognitive impairment in kindled rats. Our findings are consistent with those of previous studies showing the protective effects of sesamol against diabetes- and 3-nitropropionic acid-induced cognitive deficits (12, 13). The anti-seizure and free radical scavenging activities of sesamol may be, at least partly, involved in its ameliorative effects against cognitive impairment. In addition, sesamol down-regulates the expressions of apoptotic mark-ers including p53, Bax and caspase-3 (32), reduces the level of *mitochondrial dysfunction* *and* lipid pero-xidation *(*13*, *33*),* exerts high superoxide and NO scavenging activities (34) and therapeutic potential against chronic stress and glioma by reducing oxide-tive damage (8, 35). These mechanisms may contri-bute to the protective effects of sesamol against cognitive decline.

As previously reported, kindled seizures result in neuronal damage in the limbic system including the hippocampus and amygdala (36) that may be related to *memory loss or dysfunction*. Therefore, the limbic system may be considered as a potential target for the therapeutic action of sesamol. In addi-tion, the increased activity of glutamatergic transm-ission and formation of free radicals play a pivotal role in memory loss (37, 38), hence, it is possible that sesamol by reduction of glutamatergic neurotran-smission and formation of free radicals in the brain prevents neuronal loss and memory decline. 

As shown in Figure 6, PTZ-induced kindling elevated the level of brain MDA, an indicator of free radical generation and oxidative stress. Since oxide-tive stress has been suggested to contribute to the pathophysiology of epilepsy as well as the subseq-uent cognitive decline (15), therefore, prevention of kindling-induced MDA enhancement by seamol (30 mg/kg) pretreatment (Figure 6), suggests the antioxidant effect of sesamol as well as its protective effect against seizure-induced memory impairment. Brain GSH content was remarkably reduced following PTZ kindling (Figure 7), indicating the occurrence of oxidative stress. *GSH by scavenging* *of free radicals,* plays a pivotal role in the protection of cells against oxidative damage (13), therefore, the increased level of GSH by sesamol (30 mg/kg) (Figure 7), demonstrates the antioxidant property of this phytochemical. 

## Conclusion

Sesamol exerts ameliorative effects against seizures, cognitive impairment and oxidative stress in the experimental model of epilepsy. These findings represent sesamol as a beneficial adjuvant for antiepileptic drugs.
